# Retraction Notice

**Published:** 2011

The following article was found to be a redundant publication and is retracted herewith with this notice.

Safaei AA, Maleknejad S. Clinical and laboratory findings and therapeutic responses in children with nephrotic syndrome. Indian J Nephrol 2010;20:68-71.

**Editor, Indian Journal of Nephrology**

